# The Effects of SGLT2 Inhibitors on Blood Pressure and Other Cardiometabolic Risk Factors

**DOI:** 10.3390/ijms252212384

**Published:** 2024-11-18

**Authors:** Alexandra Katsimardou, Panagiotis Theofilis, Aikaterini Vordoni, Michael Doumas, Rigas G. Kalaitzidis

**Affiliations:** 1 2nd Department of Internal Medicine, 401 General Military Hospital of Athens, 11525 Athens, Greece; akatsimardou97@gmail.com (A.K.); michalisdoumas@yahoo.co.uk (M.D.); 2 2nd Propedeutic Department of Internal Medicine, Aristotle University of Thessaloniki, General Hospital “Hippokration”, 54642 Thessaloniki, Greece; 3 Center for Nephrology “G. Papadakis”, General Hospital of Nikaia-Piraeus “Ag. Panteleimon”, 18454 Nikaia, Greece; panos.theofilis@hotmail.com (P.T.); katerinavord@gmail.com (A.V.)

**Keywords:** hypertension, SGLT2 inhibitors, type 2 diabetes mellitus, heart failure, chronic kidney disease

## Abstract

Beyond their established hypoglycemic, cardioprotective, and nephroprotective properties, sodium–glucose cotransporters 2 (SGLT2) inhibitors exert other pleiotropic actions on blood pressure levels, body weight, and lipid metabolism. Blood pressure (BP) reduction varies based on the background history, including an effect on systolic, diastolic BP, and 24 h BP measurements. The reduction in body weight between 1 and 2 kg for the first months is caused by a reduction in visceral and subcutaneous fat due to glycosuria and loss of calories. Regarding lipid metabolism, a reduction in triglycerides and an increase in total cholesterol, high-density lipoprotein (HDL), and low-density lipoprotein (LDL) have been reported, although these alterations are small and could provide additional cardiovascular protection. Various pathophysiologic mechanisms have been proposed to explain the above-mentioned pleiotropic actions of SGLT2 inhibitors. Natriuresis, osmotic diuresis, body weight reduction, amelioration of endothelial dysfunction and arterial stiffness, sympathetic tone decrease, and uric acid reduction are among those that have been suggested for BP reduction. Apart from glycosuria and calorie loss, other mechanisms seem to contribute to body weight reduction, such as the beiging of white adipose tissue, while the mechanisms involved in lipid metabolism alterations have not been clearly determined.

## 1. Introduction

The kidney plays an important role in the action of agents that affect blood pressure (BP) levels, blood sugar levels, and body weight. Such an impressive action is attributed to the sodium–glucose cotransporters (SGTL2) as well, which are located in the S1 segment of the proximal convoluted tubule and contribute to the preservation of glucose homeostasis [1]. Under normal circumstances, approximately 180 gr of glucose is excreted in the urine daily, of which around 90% is reabsorbed through SGLT2, together with 65% of excreted sodium [2]. If plasma glucose levels rise above 180 mg/dL, which is the renal threshold for glucose reabsorption, glycosuria is observed. In patients with type 2 diabetes mellitus (T2DM), the maximum renal glucose reabsorptive capacity is increased, probably through upregulation of SGLT2, although conflicting data exist in the literature [3,4]. The increased glucose and sodium reabsorption leads to reduced delivery of sodium to the macula densa and the alteration of the tubuloglomerular feedback, resulting in vasodilation of the afferent arteriole and an increase in intraglomerular pressure [5]. This is the main proposed pathophysiologic mechanism for glomerular hyperfiltration, characteristic of the first stages of diabetic nephropathy. Thus, these important regulators of glucose homeostasis, SGLT1 and SGLT2, located in the proximal convoluted tubule, act to reabsorb the majority of glucose filtered into the glomerulus [6]. Glucose transporters (GLUTs) consequently release glucose into the circulation via the epithelial layer of the proximal tubule [7]. In patients with DM, the overexpression of GLUT genes causes an increase in SGLT2 and, therefore, increased reabsorption. The mechanisms related to the coexisting hyperglycemia are not fully understood [8]. 

The phytogenic substance phlorizin, isolated from the bark of certain plants, such as apples, provided the impetus for the development of modern synthetic SGLT2 inhibitors discovered more than 100 years ago [9]. Even though their glucosuric properties have been known for decades, it was only in 2008 that they gained approval from the U.S. Food and Drug Administration, firstly for the treatment of T2DM patients, and thereafter for other clinical conditions [9]. In terms of glycemic control, SGLT2 inhibitors are as effective as most antidiabetic agents [10].

The beneficial effects of an SGLT2 inhibitor, when added to standard care, on cardiovascular protection, heart failure, kidney protection, and death in the primary studies for the treatment of diabetes were impressive [11,12,13]. After the very encouraging results in patients with T2DM, studies in patients with heart and kidney failure showed excellent efficacy. Indeed, in patients with heart failure and a reduced ejection fraction, dapagliflozin reduced the risk of worsening heart failure or death from cardiovascular causes [14], and empagliflozin reduced cardiovascular death or hospitalization for heart failure [15]. In patients with heart failure and a preserved ejection fraction with or without diabetes, empagliflozin reduced the combined risk of cardiovascular death or hospitalization for heart failure [16], and dapagliflozin, in patients with heart failure and a mildly reduced or preserved ejection fraction, reduced the combined risk of worsening heart failure or cardiovascular death [17]. In patients with chronic kidney disease, SGLT2 inhibitors (dapagliflozin end empagliflozin) were found to exert significant renoprotective actions and are now considered the mainstay of prevention and treatment in patients with or without diabetes [18,19]. It should be noted that other SGLT2 inhibitors have demonstrated cardiovascular and renal benefits, such as ertugliflozin [20,21]. Currently, all the major guidelines support prescribing SGLT2 inhibitors, besides T2DM patients, for patients with heart failure regardless of ejection fraction (class of recommendation: I, level of evidence A), and for patients with CKD regardless of the presence of diabetes mellitus (class of recommendation: I, level of evidence A) [22,23].

Beyond their established cardio- and nephroprotective properties, SGLT2 inhibitors have other pleiotropic actions, as they seem to affect BP levels, body weight, lipid and uric acid metabolism, hematocrit, and liver steatosis. In the present narrative review, we aimed to investigate the effects of SGLT2 inhibitors as part of end-organ protection as an adjunctive BP-lowering therapy, body weight reduction, and lipid amelioration.

## 2. The Effect of SGLT2 Inhibitors on Blood Pressure

**Figure 1:**
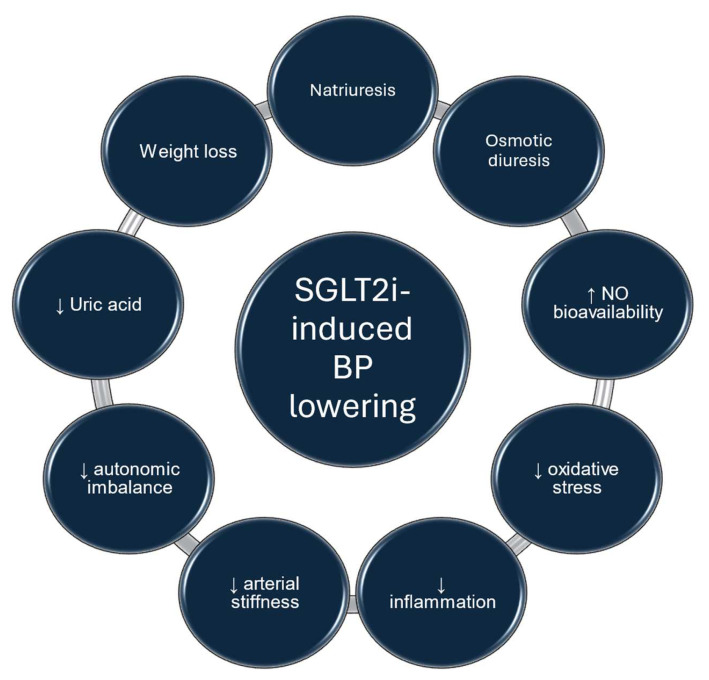
Mechanisms of SGLT2 inhibitor-induced blood pressure lowering.

Hypertension is a major risk factor for cardiovascular disease and the progression of kidney disease, notwithstanding the availability of a wide range of drugs with proven efficacy [24]. Despite the multiple antihypertensive treatments available, the achievement of BP targets is often not feasible. The recent 2023 ESH guidelines for the management of arterial hypertension clearly support the use of a RAS inhibitor as a common agent of the general combination, while the five major drug classes were recommended as first-line agents for the treatment of hypertension, i.e., angiotensin-converting enzyme inhibitors and angiotensin II receptor blockers, calcium channel blockers, Thiazide/Thiazide-like diuretics, and Beta-blockers (class of recommendation: I, level of evidence A) [22,23]. In the aforementioned guidelines, the consideration that the reduction in events due to BP lowering per se rather than to specific drug properties is further supported [25]. In the past, agents with pleiotropic actions, e.g., statins, were claimed to have antihypertensive effects [25]. Similarly, SGLT2 inhibitors and nonsteroidal mineralocorticoid receptor antagonists may also exert BP-lowering effects (Figure 1).

The benefits of SGLT2 inhibitors on BP have been evident since the first studies conducted in diabetic patients. The EMPA-REG BP trial evaluated the effect of empagliflozin on the BP of diabetic patients with stage 1 hypertension after 12 weeks of treatment. A significant reduction in 24 h systolic BP (SBP) (−3.44 mmHg, 95% CI: −4.78 to −2.09 for empagliflozin 10 mg, −4.16 mmHg, 95% CI: −5.5 to −2.83 for empagliflozin 25 mg) and diastolic BP (DBP) (−1.36 mmHg, 95% CI: −2.15 to −0.56 for empagliflozin 10 mg, −1.72 mmHg, 95% CI: −2.51 to −0.93 for empagliflozin 25 mg) was observed [26]. Similarly, in a phase 3 double-blind, placebo-controlled study, which enrolled patients from 311 centers in 16 countries, treatment with 10 mg of dapagliflozin in patients with uncontrolled T2DM and SBP and DBP values of 140–165 mmHg and 85–105 mmHg, respectively, resulted in a reduction in SBP by 4.28 (95% CI: −6.54 to −2.02) mmHg. This observed reduction in SBP was greater in those patients who were concomitantly treated with a b-blocker or a calcium channel blocker compared to those who already received a thiazide diuretic [27].

One of the first meta-analyses investigating the antihypertensive effect of SGLT2 inhibitors specifically in T2DM patients showed that they exerted a reduction in SBP and diastolic BP (DBP) by 4 (95% CI: −4.4 to −3.5) mmHg and 1.6 (95% CI: −1.9 to −1.3) mmHg, respectively. This observed reduction was evident regardless of the study type or the administered SGLT2 inhibitor [28]. In another, more recent network meta-analysis where the effects of all available antidiabetic medications on BP were assessed, SGLT2 inhibitors reduced SBP and DBP by −2.89 (95% CI: −3.37 to −2.40) mmHg and −1.44 (95% CI: −1.68 to −1.2) mmHg, respectively, while among SGLT2 inhibitors, the strongest antihypertensive properties were documented for canagliflozin and empagliflozin for SBP and for empagliflozin, dapagliflozin, and canagliflozin for DBP [29]. When compared to the other antidiabetic drug classes, SGLT2 inhibitors had the strongest antihypertensive action [28].

SGLT2 inhibitors exert a statistically significant reduction in the 24 h ambulatory BP of diabetic patients [30]. Specifically, SGLT2 inhibitors reduced the 24 h ambulatory SBP and DBP by 3.62 (95% CI: −4.29 to −2.94) mmHg and 1.7 (95% CI: −2.13 to −1.26) mmHg, respectively. The reductions in daytime SBP and DBP were −4.32 (95% CI: −5.06 to −3.57) mmHg and −2.03 (95% CI: −2.53 to −1.53) mmHg, and in nighttime SBP and DBP were −2.62 (95% CI: −3.46 to −1.78) mmHg and −1.39 (95% CI: −1.96 to −0.81) mmHg, respectively. The same meta-analysis also found that the 24 h antihypertensive effect was similar regardless of SGLT2 inhibitor dose (low or full dose) and comparable to that of low-dose hydrochlorothiazide [30].

Besides T2DM, SGLT2 inhibitors are currently prescribed for other medical conditions [22,31]. A recent meta-analysis sought to examine the effect of SGLT2 inhibitors on various cardiometabolic risk factors in patients without DM. Among the studies included in the meta-analysis, three referred to patients with heart failure [32]. A statistically significant reduction, although to a lesser extent, was observed in SBP [−1.9 (95% CI: −3.69 to −0.11) mmHg], but not in DBP [32]. Similarly, in another recent meta-analysis concerning patients with heart failure, SGLT2 inhibitors exerted a borderline reduction in SBP [−1.68 (95% CI: −2.7 to −0.66) mmHg], without significant alterations in DBP, while the heart rate of patients remained unchanged. Moreover, a statistically significant rise in plasma hematocrit [1.63 (95% CI: 0.63 με 2.62) %] was observed [33].

Even though the hypoglycemic action of SGLT2 inhibitors subsides as the glomerular filtration rate (GFR) reduces, the antihypertensive effect remains and is possibly enhanced in parallel with GFR reduction [34]. Specifically, results from a pooled analysis of phase 3 trials showed that treatment with 25 mg of empagliflozin for 24 weeks in T2DM patients resulted in a significant reduction in SBP regardless of GFR, while the biggest reduction was documented in those with the lowest GFR. 

The above-mentioned results in combination with other data from published trials regarding the effects of SGLT2 inhibitors on BP levels of different patient groups are depicted in Table 1.

## 3. The Effect of SGLT2 Inhibitors on Body Weight

As for their effect on body weight, according to a meta-analysis, a reduction of 1.85 (95% CI: −2.13 to −1.58) kg was observed in the SGLT2 inhibitor-treated group compared to the control group, while only glucagon-like peptide 1 (GLP-1) receptor agonists resulted in a greater reduction in body weight, compared to the other antidiabetic drug classes [29]. In another meta-analysis, patients’ body weight was reduced by 1.9 (95% CI: −2.5 to −1.2) kg after treatment with an SGLT2 inhibitor [28].

Moving to non-diabetic patients, a statistically significant reduction, although to a lesser extent, was observed in body weight according to another meta-analysis [−1.21 (95% CI: −1.82 to −0.61) kg] [32]. In patients with heart failure, a recent meta-analysis involving studies of this group of patients demonstrated a statistically significant body weight reduction of −1.36 (95% CI: −1.68 to −1.03) kg [33]. Finally, as far as patients with CKD of all stages are concerned, a reduction in body weight was similarly evident [34].

## 4. Pathophysiologic Mechanisms Involved in the BP-Lowering Effect of SGLT2 Inhibitors

Natriuresis and osmotic diuresis with contraction of extracellular fluid, which have been confirmed as mechanisms of action of SGLT2 inhibitors, could be responsible for the BP-lowering effect. Other mechanisms have also been proposed including the improvement in endothelial function, improvement in arterial stiffness, reduction in sympathetic nervous system activity, renin–angiotensin–aldosterone system (RAAS) activation, and, finally, serum uric acid reduction [39,48,49,50,51].

The inhibition of glucose and sodium reabsorption results in glycosuria, natriuresis, and osmotic diuresis. The increased sodium delivery to the macula densa restores the tubuloglomerular feedback, leading to afferent arteriolar vasoconstriction, while the inhibition of renin release from the juxtaglomerular cells leads to efferent arteriolar vasodilation, resulting in the inhibition of hyperfiltration, the reduction in intraglomerular hydrostatic pressure, and the amelioration of the inflammatory and fibrotic changes that take place in the diabetic kidney [48]. A clinical consequence of this phenomenon is the observed initial drop in GFR at the start of pharmacotherapy, which is, however, fully reversible after drug discontinuation, simulating the changes in GFR seen at the start of RAAS inhibitor treatment [49].

The inhibition of the renal sodium–hydrogen exchanger 3 contributes to the natriuretic action of SGLT2 inhibitors [50]. However, recent evidence has shown that the observed natriuresis is transient and gradually subsides, probably due to sodium reabsorption in the more distal tubular segments. In particular, in a study among normotensive, non-diabetic individuals, 180 min after the ingestion of a single dose of empagliflozin, an increase in the fractional excretion of sodium by 35.6% was observed. A month later, however, the fractional excretion of sodium had subsided to 8%. Meanwhile, increases in plasma renin and aldosterone concentrations were found after a month of empagliflozin treatment, indicating RAAS activation. Despite these observations, data from 24 h ambulatory BP measurements showed that a statistically significant reduction in SBP and DBP by 5 mmHg and 2 mmHg, respectively, remained [52]. These data, combined with the antihypertensive effect of these drugs in patients with low eGFR, where these drugs do not have a satisfactory natriuretic and hypoglycemic effect, suggest the existence of other mechanisms in the reduction in BP.

The effect of SGLT2 inhibitors on the endothelium has been the subject of investigation in a limited number of studies in patients with DM. Results from a recent meta-analysis of studies that assessed the effect of dapagliflozin on flow-mediated dilation (FMD) showed improvement in FMD values, further confirming the favorable effect of dapagliflozin on the endothelium [increase in FMD by 1.66% (95% CI: 0.56 to 2.76, I^2^ = 28%, *p* = 0.003)] [53]. Meanwhile, several data show that SGLT2 inhibitors positively affect arterial stiffness through reductions in pulse wave velocity [39,51]. In light of these issues, SGLT2 inhibitors restore autophagy, which also may help to reduce oxidative stress, inflammation, and endothelial dysfunction in experimental models [54]. SGLT2 inhibitors improve inflammatory mechanisms such as NLRP3 inflammasome and nuclear factor kB [55].

Similarly, SGLT2 inhibitors improve nitric oxide (NO) bioavailability and reduce endothelial cell apoptosis, possibly due to increased activation of the Akt/endothelial NO synthase pathway [56], increased adenosine monophosphate-activated protein kinase (AMPK) [57], activated potassium channels and protein kinase-G [58], and improved erythropoiesis [59].

SGLT2 inhibitors also exhibit renoprotective effects via their effects on inflammation, apoptosis, angiogenesis, and fibrosis in early-stage diabetic nephropathy, with improvements in histopathological examinations, apoptosis markers B-cell lymphoma (BCL-2) and BCL2-associated X (BAX) [60]. The aforementioned mechanisms not only improve kidney function but also induce BP lowering. Additionally, SGLT2 inhibitors reduce glomerular and interstitial fibrosis, and this could have an effect on improving volume dependence, reducing peripheral resistances, and BP levels [61,62]. In animal experiments, SGLT2 inhibitors reduce inflammation and enhance mitochondrial function through AMPK-OPA1 pathway promotion by reversing mitochondrial division and protecting against renal ischemia–reperfusion injury [63].

Finally, the same anti-atherosclerotic actions through the pleiotropic mechanism of SGLT2 inhibitors could actively contribute to the reduction in vascular resistance and BP levels due to their effect on inflammation [64], oxidative stress [65], endothelial dysfunction [64], and platelet activation [66]. On top of these, restriction of growth and migration of vascular smooth muscle cells by heme oxygenase could also be involved in the anti-atherosclerotic actions of these drugs [66,67], which in turn could improve peripheral vascular resistance and BP levels.

It is very interesting that experimental data have shown that SGLT2 inhibitors also affect the sympathetic nervous system, providing another possible explanation for their antihypertensive properties. There is evidence for significant crosstalk between the activation of the sympathetic nervous system and SGLT2 regulation and possible ancillary effects on endothelial function. Studies from experimental animal models proved that dapagliflozin decreased tyrosine hydroxylase expression and norepinephrine levels in the kidneys, exerting clinical results similar to those observed after chemical denervation of the sympathetic nervous system, such as a decrement in BP levels and improvement in glucose homeostasis [68]. Finally, another important hypothesis is whether body weight reduction may have an effect on BP reduction. Even though there is a positive correlation between body weight with BP, this effect seems to be small [69,70].

## 5. Proposed Pathophysiologic Mechanisms Involved in Body Weight Reduction Though SGLT2 Inhibition

Glycosuria leads to forced weight loss. Induced glycosuria corresponds to 240–320 loss of calories per day. Body weight is stabilized after the first months of treatment despite ongoing glycosuria, possibly in the context of compensatory overeating and higher calorie consumption [71]. Short-term contraction of extracellular volume due to osmotic diuresis also contributes to body weight loss. This negative caloric balance accounts for visceral fat and liver steatosis amelioration, where two-thirds of the observed reduction in body weight is due to losses from adipose tissue, with a slight predominance of losses from visceral over subcutaneous fat [72].

Recent experimental data show that SGLT2 inhibitors affect adipose tissue by other mechanisms as well. SGLT2 inhibitors are a promising strategy to combat the growing epidemic of obesity by inducing browning/beiging of white adipose tissue (WAT). Studies in experimental animals showed that the administration of dapagliflozin increased the concentration of tyrosine hydroxylase and norepinephrine in adipose tissue, while at the same time, a statistically non-significant increase was observed in the expression of the uncoupling protein Ucp1 and the transcription factor Pgc-1a, in the context of grey adipogenesis [73]. Similarly, empagliflozin also increased the expression of uncoupling protein 1 in brown fat and in inguinal and epididymal WAT, which resulted in heat production and energy expenditure [74]. Additionally, it has been shown that increased energy consumption stimulated by chronic canagliflozin use leads to greater intra-adipose sympathetic innervation, ultimately ameliorating diet-induced obesity [75].

Recent studies using animal experiments showed that dapagliflozin promotes the browning of WAT through fibroblast growth factor receptors 1(FGFR1), liver kinase B1 (LKB1), and AMPK signaling pathway [76]. Dapagliflozin could also reduce body weight gain by regulating lipogenesis and angiogenesis in WAT. Indeed, dapagliflozin prevents cellular differentiation and upregulates the expression of WAT browning and angiogenesis genes in 3T3-L1 adipocytes in vitro [77].

In light of these issues, empagliflozin administration also suppressed high-fat diet-induced weight gain by shifting energy metabolism towards fat utilization, elevating AMP-activated protein kinase, phosphorylation of the acetyl-CoA carbolxylase in skeletal muscle, and increasing hepatic and plasma FGF21 levels [74]. Other proposed mechanisms suggest that empagliflozin enhanced fat utilization and browning, attenuated obesity-induced inflammation, and suppressed weight gain and insulin resistance by polarizing M2 macrophages and lowering plasma tumor necrosis factor-alpha (TNFα) levels in WAT and the liver [74,78]. Finally, in animal experiments, empagliflozin influenced hypothalamic energy homeostasis, acting as a potential regulator in obesity by directly influencing the expression of endogenous mRNA of pro-opiomelanocortin (POMC) and agouti-related peptide (AgRP), which are critical for energy homeostasis and modulate their transcription in high-fat diet-induced obesity [79].

As it becomes evident, there are several mechanisms that have been implicated in SGLT2 inhibitor-induced weight loss. However, since numerous factors are involved in the weight control of diabetic patients, further research is required to confirm the exact pathways involved.

## 6. Effect of SGLT2 Inhibitors on Lipid Metabolism

Several lines of evidence support that SGLT2 inhibitors may play a cardioprotective role in dyslipidemia in diabetic patients [80]. Data from a recent meta-analysis of 48 randomized trials showed that the administration of SGLT2 inhibitors resulted in a reduction in triglyceride levels (−0.10 mmol/L, 95% CI: −0.13 to −0.07) and an increase in total cholesterol levels (0.09 mmol/L, 95% CI: 0.05 to 0.13) in low-density lipoprotein (LDL) (0.10 mmol/L, 95% CI: 0.07 to 0.12) and in high-density lipoprotein (HDL) (0.06 mmol/L, 95% CI: 0.05 to 0.08), without affecting the LDL-to-HDL ratio. The largest increase in LDL was observed with canagliflozin (approximately 5 mg/dL) and the largest increase in total cholesterol with empagliflozin (4.25 mg/dL) [81]. A recent meta-analysis included 60 randomized trials, with 147,130 individuals. Even in this meta-analysis, SGLT2 inhibition increased total, LDL cholesterol, and HDL cholesterol, and decreased triglycerides [82]. Meanwhile, new players are entering the SGLT2 market. In a recent meta-analysis on remogliflozin, a novel SGLT2 inhibitor, a significant increase was also found in HDL cholesterol levels [83].

The causes of these changes are not precisely ascertained. Among the various hypotheses, it has been suggested that there is increased expression and activity of the enzyme 3-hydroxy-3-methylglutaryl-coenzyme A reductase, low expression of the LDL receptor in the liver parenchyma, and reduced uptake of LDL by hepatocytes [84]. At the same time, however, it seems that qualitative changes in LDL cholesterol are also induced. The administration of dapagliflozin for 12 weeks in patients with T2DM, although not affecting total LDL cholesterol levels, reduced the concentration of small dense atherogenic LDL particles by 20% and increased the concentration of larger LDL particles by 18%. Similar findings were reported in the subgroup of those in whom an increase in LDL cholesterol concentration was observed [85]. The above changes, however, do not translate into cardiovascular benefit or risk according to the data so far, as further studies are needed for this purpose.

Ιndirect indications for the beneficial action of SGLT2 inhibition come from studies in patients with metabolic dysfunction-associated steatotic liver disease (MASLD) and an increased atherogenic profile. Ιn a recent retrospective longitudinal study on patients with T2DM and MASLD, three-year SGLT2 inhibitor treatment significantly reduced LDL cholesterol, triglyceride, and non-HDL cholesterol, suggesting that SGLT2 inhibitors may be an ideal therapy for MASLD patients with atherosclerosis [86]. Given that SGLT2 inhibitors contribute to the observed lower cardiovascular risk, we can make some assumptions about the effectiveness of SGLT2 inhibitors. SGLT2 inhibitors may exhibit potential direct and indirect cardiovascular benefits. They act directly on cardiomyocytes and endothelial cells and lower intracellular sodium and calcium levels [50], inhibit vascular smooth muscle cell proliferation and migration [66], reduce inflammation, mitigate oxidative stress and endoplasmic reticulum (ER) stress [65], and regulate autophagy [55,64], angiogenesis, and apoptosis [60]. Furthermore, SGLT2 inhibitors suppress the expression and activity of hypoxia-inducible factor 1-alpha (HIF-1α) [87], which may enhance atherosclerotic plaque instability and promote cardiac fibrosis [88]. Indeed, dapagliflozin attenuates cardiac fibrosis and inflammation by reverting the HIF-2a signaling pathway in arrhythmogenic cardiomyopathy [88]. On the other hand, the indirect effect could be attributed to the improvement in cardiac function [55], preservation of kidney function [61,62,63], BP control [35,43], and alleviation of anemia [89], factors associated with better clinical outcomes.

## 7. Conclusions

In summary, SGLT2 inhibitors modestly reduce BP in diabetic and non-diabetic patients by approximately 3–5 mmHg, which varies slightly by specific SGLT2 inhibitor but does not reach levels typically achieved by primary antihypertensive agents. These agents reduce body weight through a reduction in visceral and subcutaneous fat, while it seems that their effect on the lipid profile could provide additional cardiovascular protection in the long term. Their metabolic benefits cannot be interpreted only through induced glycosuria and natriuresis, while the antihypertensive mechanism of SGLT2 inhibitors is multifactorial. Ιt is impressive that first we discovered their therapeutic properties and then we tried to focus on their multiple mechanisms of action. It should be acknowledged that further data are needed to elucidate the underlying still unknown pathophysiological mechanisms.

## Figures and Tables

**Table 1:** Effect of SGLT2 inhibitors on blood pressure.

Author/Study Design	Population	Ν	BP Assessment	Intervention vs. Control	Study Duration	Change in SBP/DBP
Non-diabetic patients						
Zanchi et al. [35] Double-blind RCT	Normotensive non-diabetic patients	39	24 h ABPM	10 mg empagliflozin vs. placebo	1 m	Change in 24 h SBP/DBP: Empagliflozin: −5/−2 Placebo: 3/2 Change in daytime SBP/DBP: Empagliflozin: −4/−1 Placebo: 1/1 Change in nighttime SBP/DBP: Empagliflozin: −6/−4 Placebo: 5/1
Teo et al. [32] Meta-analysis of eight RCTs	Non-diabetic patients	5233	Office BP	Dapagliflozin, empagliflozin, canagliflozin vs. control group	12 w–18 m	Change in SBP/DBP vs. control: −1.9/0.27
Patients with T2DM and hypertension						
Kario et al. [36] SACRA study Multicenter double-blind RCT	T2DM patients under stable antihypertensive treatment including an ARB and uncontrolled nocturnal HTN	132	24 h ABPM	10 mg empagliflozin vs. placebo	12 w	Between-group differences: 24 h SBP/DBP: −7.7/−2.9 (*p* < 0.05) Daytime SBP/DBP: −9.5/−3.9 (*p* < 0.05) Nighttime SBP/DBP: −4.3/−1.6 (*p* > 0.05)
Kario et al. [37] SHIFT-J study Open-label RCT	T2DM patients with uncontrolled HTN under stable antihypertensive treatment	84	Office and home BP	100 mg canagliflozin vs. control group	8 w	Change in nighttime home SBP: Canagliflozin: −5.23 Control group: −1.04 (*p* = 0.078) Change in evening home SBP: Canagliflozin: −8.7 Control group: 2.4 (*p* = 0.012)
Ferdinand et al. [38] Double-blind RCT	Black T2DM patients with uncontrolled HTN	150	Office BP and 24 h ABPM	25 mg empagliflozin vs. placebo	24 w	Between-group differences: 24 h SBP: −8.39 Office SBP/DBP: −7.43/−4.25
Tikkanen et al. [26] Double-blind RCT	T2DM patients with stage 1 HTN	825	Office BP and 24 h ABPM	Empagliflozin 10 mg and 25 mg vs. placebo	12 w	Between-group difference in 24 h SBP/DBP: −3.44/−1.36 for empagliflozin 10 mg, −4.16/−1.72 for empagliflozin 25 mg Between-group difference in office SBP/DBP: −3.92/−1.93 for empagliflozin 10 mg, −4.8/−1.89 for empagliflozin 25 mg
Patients with T2DM						
Papadopoulou et al. [39] Double-blind RCT	T2DM patients	85	24 h ABPM	Dapagliflozin 10 mg vs. placebo	12 w	24 h SBP/DBP: Dapagliflozin: −5.8/−2.2 Placebo: −0.1/0.1 Central 24 h SBP: Dapagliflozin: −4.1 Placebo: −0.7
Kario et al. [40] LUSCAR Study Multicenter trial	T2DM patients	47	Office and home BP	Luseogliflozin 2.5 mg	12 w	Change in morning home SBP/DBP: −5.2/−2.5 Change in evening home SBP/DBP: −5.5/−2.9
Saito et al. [41] PROTECT study Multicenter open-label RCT	T2DM patients	232	Office BP	Ipragliflozin 50–100 mg vs. control group	24 m	Between-group difference in SBP: −3.6 mmHg (−6.2 to −1 mmHg)
Baker et al. [28] Meta-analysis of 27 RCTs	T2DM patients	12.960	Office BP	Canagliflozin Dapagliflozin Empagliflozin Ipragliflozin Remogliflozin vs. placebo (21 studies) or other antidiabetic treatment (6 studies)	3–52 w	Mean between-group difference in SBP/DBP: −4/−1.6
Tsapas et al. [29] Meta-analysis of 204 RCTs	T2DM patients	165.639	Office BP	SGLT2i vs. control group	>24 w	Between-group difference in SBP/DBP: −2.89/−1.44
Georgianos et al. [30] Meta-analysis of seven RCTs	T2DM patients	2.381	24 h ABPM	Canagliflozin Dapagliflozin Empagliflozin Ertugliflozin vs. placebo (five studies) and low-dose Hydrochlorothiazide (two studies)	4–12 w	Between-group difference in 24 h SBP/DBP: −3.62/−1.7 Between-group difference in daytime SBP/DBP: −4.32/−2.03 Between-group difference in nighttime SBP/DBP: −2.62/−1.39
Patients with CKD						
Ye et al. [42] CREDENCE study Multicenter double-blind RCT	Patients with T2DM and CKD (eGFR 30–90 mL/min/1.73 m^2^ and UACR 300–5000 mg/dL)	4401	Office BP	100 mg canagliflozin vs. placebo	2.6 y	Between-group difference in office SBP: −3.3 (*p* = 0.84)
Provenzano et al. [43] DAPA-CKD study Double-blind RCT	Patients with CKD (eGFR 25–75 mL/min/1.73 m^2^ and UACR 200–5000 mg/dL)	4304	Office BP	Dapagliflozin 10 mg vs. placebo	2.4 y	Between-group difference in office SBP: −2.9 mmHg
Mayne et al. [44] EMPA-KIDNEY study Double-blind RCT	Patients with CKD (eGFR 20–45 mL/min/1.73 m^2^ or 45–90 mL/min/1.73 m^2^ and UACR 200 mg/dL)	6609	Office BP	Empagliflozin 10 mg vs. placebo	2 y	Between-group difference in office SBP/DBP: −2.6/−0.5
Barnett et al. [45] EMPAREG RENAAL study Multicenter double-blind RCT	Patients with T2DM and CKD stages 2 and 3	290 with stage 2 CKD, 374 with stage 3 CKD, 74 with stage 4 CKD	Office BP	Empagliflozin 10 mg and 25 mg vs. placebo	52 w	With stage 2 CKD: Between-group difference in SBP/DBP: −3.3/−2.7 for empagliflozin 10 mg, −7.8/−4.5 for empagliflozin 25 mg With stage 3 CKD: Between-group difference in SBP/DBP: −4.3/−1.5 for empagliflozin 25 mg With stage 4 CKD: Between-group difference in SBP/DBP: −11.2/−4.3 for empagliflozin 25 mg
Kinguchi et al. [46] Y-AIDA study Prospective multicenter study	Patients with T2DM, eGFR ≥ 45 mL/min/1.73 m^2^ and UACR ≥30 mg/g creatinine	86	Home BP	Dapagliflozin 10 mg	24 w	Change in morning SBP/DBP: −8.32/−4.18, *p* = 0.001 Change in evening SBP/DBP: −9.57/−4.48, *p* = 0.001 Change in nighttime SBP/DBP: −2.38/−1.17, *p* < 0.05
Patients with heart failure						
Li et al. [33] Meta-analysis of 16 RCTs	Patients with HFrEF (eight studies), HFpEF (four studies) or with HF regardless of EF (four studies)	7.696	Office BP	Dapagliflozin Empagliflozin Canagliflozin Luseogliflozin or placebo	6 w–26.2 m	Change in SBP: −1.68 for SGLT2i
Chatur et al. [47] DELIVER study Double-blind RCT	Symptomatic HF with EF > 40% and hospitalization in the previous 30 days for decompensated HF	654	Office BP	Dapagliflozin 10 mg vs. placebo	1 m	Placebo: +1.4 mmHg Dapagliflozin: +0.2 mmHg Between-group difference: −1.3 mmHg (−3.6 to 0.9)
